# Recurrent Stroke Events Secondary to a Late Presentation of Mitochondrial Encephalomyopathy With Lactic Acidosis and Stroke-Like Symptoms (MELAS) Syndrome

**DOI:** 10.7759/cureus.11839

**Published:** 2020-12-02

**Authors:** Ihab Jameel, Abuajela Sreh, Partha Das

**Affiliations:** 1 Internal Medicine, University Hospitals of Derby and Burton NHS Foundation Trust, Derby, GBR; 2 Gastroenterology, Barnsley Hospital NHS Foundation Trust, Barnsley, GBR; 3 Stroke/Geriatrics, University Hospitals of Derby and Burton, Derby, GBR

**Keywords:** recurrent strokes, cva, cerebrovascular accident, mitochondrial, melas, siadh

## Abstract

Mitochondrial encephalomyopathy with lactic acidosis and stroke-like symptoms (MELAS) is a rare mitochondrial disorder that typically presents before the age of 40 with most patients diagnosed before the age of 20. Symptoms and signs typically include mitochondrial myopathy, encephalopathy with stroke-like episodes, seizures and/or dementia, and lactic acidosis. We present a case of a 56-year-old lady presenting with recurrent ischaemic strokes and seizures associated with non-territorial low attenuation areas on brain imaging. Together with a raised serum lactate and background history of Syndrome of Inappropriate secretion of Anti-Diuretic Hormone (SIADH), genetic analysis was carried out that confirmed the presence of the most common mutation associated with MELAS syndrome which is m.3243A>G mutation. This case raises the importance of considering a diagnosis of inherited mitochondrial disorder when faced with recurrent atypical stroke-like episodes, when neuro-imaging is inconsistent with ischemic infarction, even in adults or elderly individuals. It also highlights the importance of background history and associated conditions that should be put into consideration when thinking about differential diagnosis.

## Introduction

The clinical syndrome of MELAS (mitochondrial encephalomyopathy, lactic acidosis, and stroke-like episodes) is caused by mutations in mitochondrial deoxyribonucleic acid (DNA) and subsequent respiratory chain deficiency. Symptoms and signs typically comprise mitochondrial myopathy, encephalopathy with stroke-like episodes, seizures and/or dementia, and lactic acidosis [1,2]. SIADH or renal impairment have been identified as possible causes of hyponatremia in MELAS [3,4]. Since stroke-like episodes (SEs) are similar to acute cerebral infarction, they are often misdiagnosed likely as they are difficult to recognize and extremely rare in the general population. We present a case of a 56-year-old lady presenting with recurrent ischaemic strokes and seizures associated with non-territorial low attenuation areas on brain imaging.

## Case presentation

A 56-year-old lady was brought into the hospital by ambulance with a non-specific headache, flashing lights, and blurred vision. She denied any history of limb weakness, sensory deficit, speech disturbance, or balance problems. She had no chest pain, shortness of breath, or dizziness. The patient denied any abdominal pain, vomiting, or urinary symptoms. Also, there was no fever or weight loss. She had a history of essential Hypertension, Type two diabetes mellitus, hypercholesterolemia, hearing loss, and Syndrome of Inappropriate secretion of Anti-Diuretic Hormone (SIADH). Her comorbidities were generally controlled with medications. She never smoked in her life and consumed about 3-4 drinks per week. The patient was on paracetamol, atorvastatin, clopidogrel, amlodipine, cyclizine, and insulin and had no known allergies. She is independent and lives with her husband and children. 

On examination, the patient was alert and oriented. Vital signs were within the normal range. Auscultation revealed normal heart sounds with no murmurs and bilateral equal air entry with no crackles. The abdomen was soft and non-tender, and bowel sounds were present. The calves were soft and non-tender, peripheral pulses were intact, and no pedal edema. Neurological examination revealed right homonymous hemianopia, otherwise normal upper and lower limb examination, normal cranial nerve examination, and normal cerebellar signs. 

**Figure 1:**
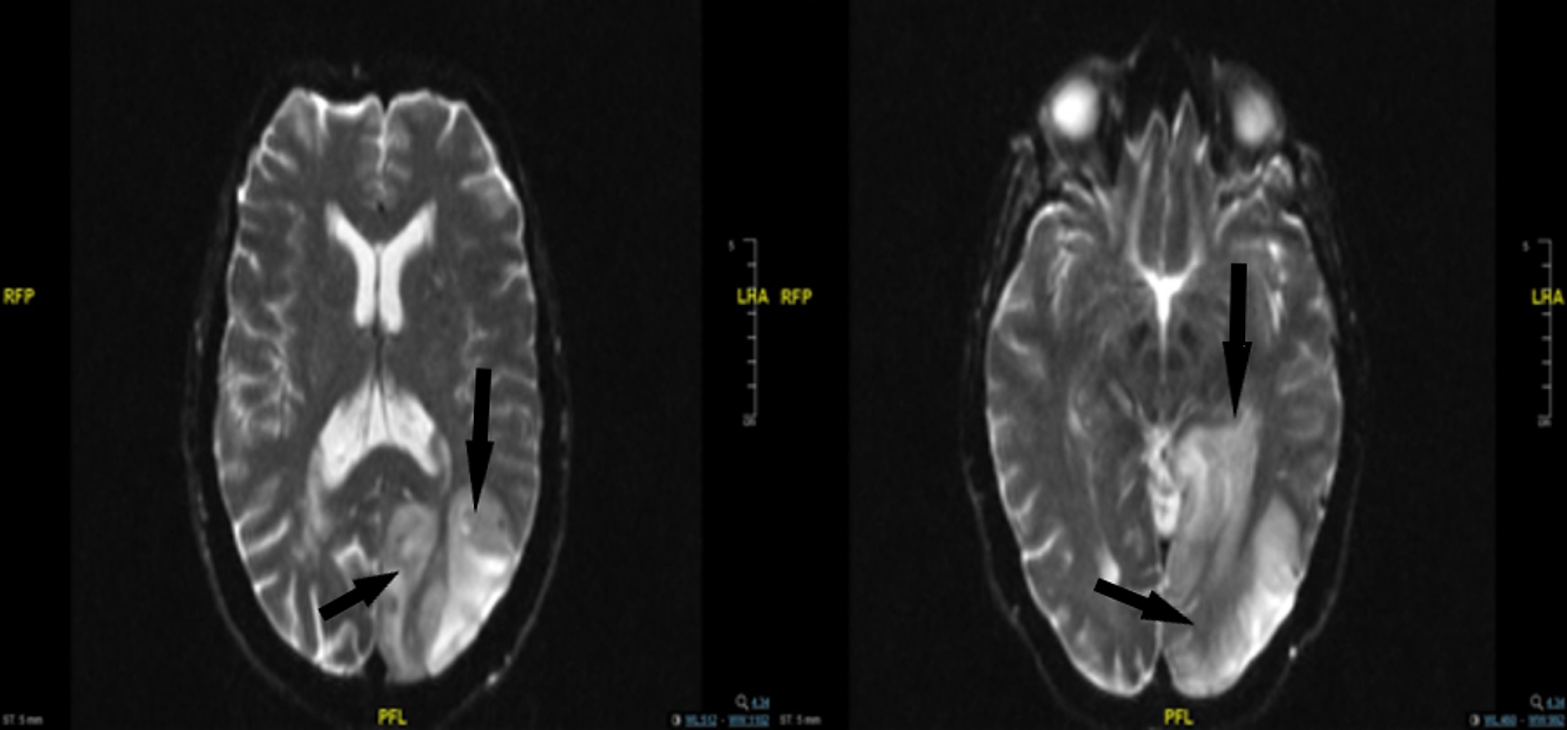
Ep2d diffusion axial MRI head showing a large left occipito-parietal gyriform cortical area of restricted diffusion (black arrows)

Blood results showed a white cell count of 7.3×10^9^/L (normal 4.0-10.0×109/L) and a C-reactive protein (CRP) level of <5 mg/L (0-5). Hemoglobin was 128 g/L (120-150) with normal mean corpuscular volume (MCV). Also, her sodium was noted to be low with a level of 128 mmol/L (133-146), urea 5.4 mmol/L (0-8.3), and normal renal function tests. Glucose was 6.8 mmol/L (4.1-6) on admission. 12-lead electrocardiography (ECG) showed normal sinus rhythm. Initial computed tomography (CT) head showed a large left occipito-parietal and smaller right parietal lobe recent infarcts. No abnormality was detected in chest x-ray and no significant stenosis in the carotid doppler ultrasound study. Magnetic resonance imaging (MRI) of her head showed a large left occipito-parietal area of gyriform restricted diffusion and white matter ischemic patches (Figure 1). The patient was treated for an ischaemic stroke and started on aspirin 300 mg daily for 14 days followed by clopidogrel as per guidelines, as she was outside the thrombolysis window. An echocardiography was performed and showed normal heart structure and valves, with no evidence of patent foramen ovale and ejection fractions (EF) of 70%. Thrombophilia, Lupus, Syphilis, and Hepatitis screening were done, in addition to protein electrophoresis and beta-glycoprotein, and all were negative. She was discharged home with outpatient follow-up in stroke clinic and orthoptist. Neurologically patient had a residual mild right-sided visual defect. 

Six weeks after her discharge, she had an episode of hemiparesis in the right arm and right leg with right-sided visual neglect and mild speech disturbance. The weakness lasted for four days, and a repeated MRI head showed resolution of the previously noted infarct and no evidence of further new strokes. She was seen in the outpatient clinic four weeks later and had recovered quite well since then and noticed that her eyesight has recovered.

Two months later, the patient was admitted for the third time with an episode of tonic seizures with loss of consciousness for around four minutes and post-ictal confusion, but there was no tongue biting or loss of urinary and bowel control. CT Head showed no acute intracranial pathology. The patient was diagnosed as Post-Stroke Seizure and was started on Lamotrigine and discharged home after observation with neurology follow-up as an outpatient. After that, the patient was seen by a neurologist at an outpatient clinic, and an electroencephalogram (EEG) was ordered. No abnormality was detected, and no epileptiform features were recorded. The patient had scattered episodes of focal seizures, and her Lamotrigine dose has been adjusted multiple times by her neurologist. Given her background history of SIADH, a blood test was sent for serum lactate level as the possibility of MELAS syndrome was considered, and her serum lactate came back at 2.9 mmol/L (0-1.30 mmol/L). Therefore another blood sample was sent off for Mitochondrial Encephalopathy with Lactic Acidosis and Stroke-like episodes (MELAS) genetic test. She also had her paraneoplastic antineuronal antibodies in serum (Anti-Hu, Anti-Yo, Anti-Ri, Anti-CV2, Anti-Ma1, Anti-Ma2/Ta, Anti-Amphiphysin, Anti-VGCC, and Anti-NMDA-receptor antibodies) which were negative.

**Figure 2:**
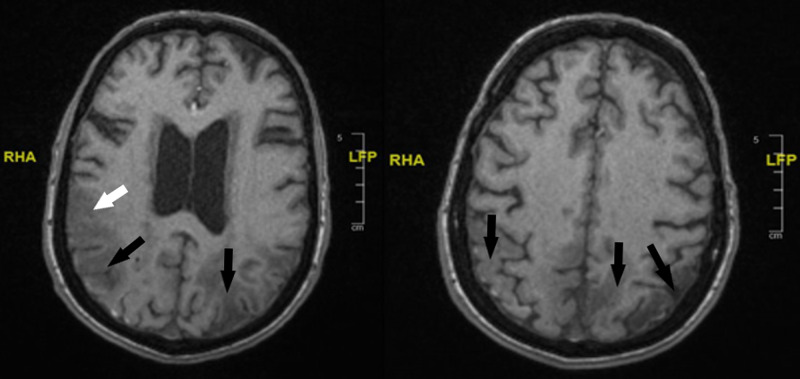
T1 axial MRI head showing widespread bright signals in the bilateral parieto-occipital regions (black arrows) and to a lesser extent involving the Temporal lobes (white arrow)

Ten months after her initial stroke, the patient was admitted for the fourth time with an episode of visual disturbance in the left eye, described as flashing light lasting for a few seconds. She denied any headache, weakness, loss of sensation, or gait problems. CT Head was done and showed a recently formed low attenuation area in the right temporal and occipital region. A repeated Carotid Doppler Ultrasound showed no significant stenosis bilaterally. Five-day ECG monitoring showed normal sinus rhythm throughout. A repeat MRI head showed acute widespread bright signals involving the right parieto-occipital region and, to a lesser extent involving the temporal lobes bilaterally with chronic left side changes as seen before (Figure 2). The patient was also fitted with a Cardiac reveal device (Implantable loop recorder) that showed no rhythm abnormality to explain her symptoms.

Nine months later, she had a fifth admission with a two-day history of recurrent and self-terminating tonic-clonic seizures, and her anti-epileptics were adjusted. At this stage, her Alpha-galactosidase A levels were normal, ruling out Fabry’s disease. Also, her genetic analysis for MELAS syndrome was positive in the form of the M.3243A > G mutation. Results and implications were explained to the patient, and she was referred to the Adult Inherited Metabolic Disease (IMD) Services as well as a clinical genetics department at a tertiary center for further management.

**Figure 3:**
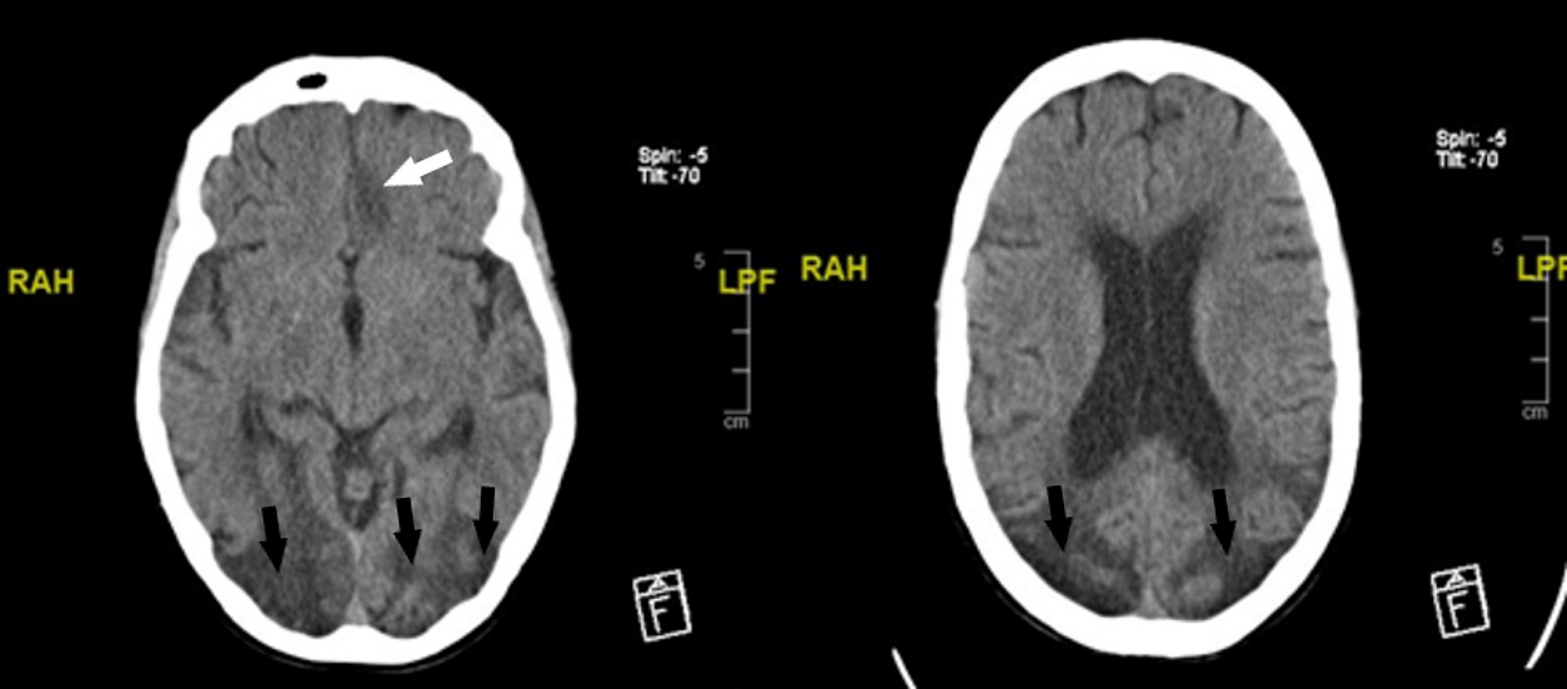
3-mm axial CT head showing bilateral parieto-occipital chronic extensive confluent low attenuation areas are more hypodense and mildly more expanded, likely due to progression of the atrophic changes (black arrows). In the left frontal lobe there is a paramedian moderately hypodense area (white arrow).

**Figure 4:**
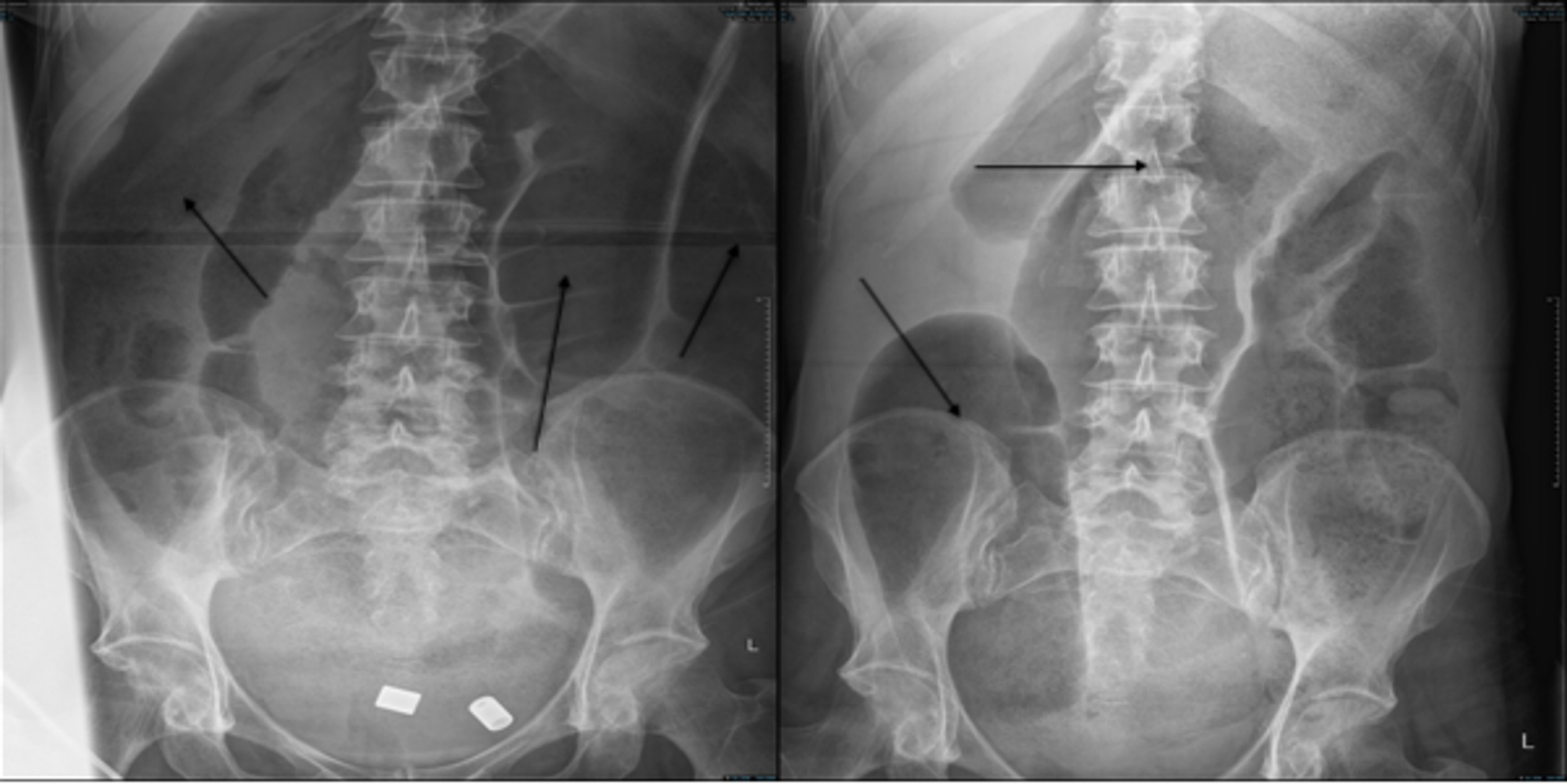
Plain abdominal X-ray showing dilated featureless loops of large bowel secondary to sigmoid volvulus

Eighteen months later, the patient was re-admitted for the sixth time with an episode of confusion and a possible unwitnessed seizure. CT head showed a subacute paramedian low attenuation area in the left frontal lobe, which previously was not seen (Figure 3). She was then transferred to a local stroke center for ongoing stroke management. Afterwards, the patient had two episodes of sigmoid volvulus and Pseudo-obstruction that required surgical intervention, which is also a rare occurrence in MELAS syndrome (Figure 4). 

## Discussion

Mitochondrial Encephalomyopathy with Lactic Acidosis and Stroke-like episodes (MELAS) syndrome is a rare genetic disorder characterized by affecting the nervous system and muscle. It is caused by a mutation in the mitochondrial DNA (mtDNA). The most common mutation associated with MELAS is the m.3243A>G mutation which is found in approximately 80% of MELAS cases [5]. The m.3243A>G mutation is also known to be associated with other clinical presentations such as maternally inherited deafness and diabetes (MIDD) and Progressive External Ophthalmoplegia (PEO) [6]. One in 400 of the m.3243A>G mutation will be a carrier. MELAS affects both genders equally, and the age of onset is usually between 2 and 15 years. Approximately 75% of the cases occur less than the age of twenty; however, a few cases are reported in the literature above the age of 50 [5-7].

Mitochondrial mutations are passed on to the next generations through female patients only. Both normal and mutated mtDNA could exist in the same cell, and accumulation of the mutated mtDNA will determine the manifestation of clinical symptoms. Hence, not all generations would show clinical features. Also, it depends on which cell type is affected; the clinical characteristics will be, therefore affected family members might exhibit different clinical features [7].

Clinically MELAS patients could present with a wide variety of symptoms, including neurological deficits resembling stroke features, headaches, particularly migrainous type headache, epileptic seizures most commonly in the form of status epilepticus, psychiatric signs, and gastrointestinal symptoms. Certain complications also were seen, such as cardiomyopathy, nephrotic syndrome, diabetes, etc. [5,7]. Seizures occur in up to 70% of MELAS patients older than 50 [5]. SIADH or renal impairment have been identified as possible causes of hyponatremia in MELAS [3,4]. In addition, hearing impairment is common in patients with mitochondrial disorders, affecting over half of all cases at some time in the course of the disease [8]. These conditions were noticed in our patient, which ultimately lead to investigating MELAS as a probable cause for her presentations.

Diagnosis of MELAS is based on a combination of clinical manifestations, raised serum or CSF lactate levels, and brain imaging. CT or MRI head typically would show focal lesions most commonly in the temporal area explaining seizure activity or stroke-like ischaemic changes that otherwise do not correlate with a specific arterial territory seen in small vessel disease. Based on clinical suspicion, the diagnosis should be confirmed by carrying out a genetic analysis for m.3243A>G mutation in blood or other tissue biopsies such as skin, urine, muscle, etc. [5,7].

There is no curative treatment for MELAS currently, and management based on the associated clinical features such as anti-platelets for ischaemic stroke episodes, anti-convulsants for MELAS related epilepsy, etc. A Cochrane review was conducted in 2012 that concluded there is no evidence to support any intervention in mitochondrial diseases; however, a few centers are using L-arginine in SEs [5,7,9].

MELAS has an annual mortality rate of 5%-8% with worse mortality in juvenile cases than when diagnosed later on in life. The medical age from diagnosis is 6.4 years in juvenile MELAS compared to 10 years in adult MELAS patients [10]. As in our patient’s MRI, MELAS lesions are typically localized in the temporo-occipital cortex and may progress over time, extending to adjacent areas without respecting arterial vascular territories [11]. Both grey and white matter are affected and appear hyperintense on FLAIR or T2w images as a sign of edema, resulting in a pronounced local mass effect.

Interestingly, our patient had changes involving all four lobes of the cerebral hemisphere, with bilateral changes in most of them, which were also unusual and further supported the diagnosis of a stroke-like condition. Also, very few articles in the literature have described frontal lobe changes in cases of MELAS syndrome. The educational value of our case lies in having a high suspicion of MELAS syndrome in a patient presenting with recurrent SEs with MRI findings that are not related to a single domain; especially if a background history of an associated condition like SIADH as in our patient is present, which have finally led to the correct diagnosis.

## Conclusions

MELAS typically presents before the age of 40; however, if clinically suspected, the genetic analysis should be carried out regardless of the age. It presents a broad spectrum of symptoms based on the organs affected, most commonly SEs, seizures, psychiatric and behavioral symptoms. Associated conditions like SIADH and gastrointestinal conditions should always be considered when making a differential diagnosis. Cochrane review conducted in 2012 confirmed no substantial evidence supporting any intervention for MELAS. However, a few reports suggest the use of L-arginine amino acid to help with Stroke-like symptoms. Certain medications are used to treat certain complications, such as anti-convulsants, to treat MELAS epileptic seizures. Hence, we recommend considering investigating rarer causes of Stroke in patients with recurrent Stroke symptoms in all age groups.

## References

[1] McFarland R, Taylor RW, Turnbull DM (2010). A neurological perspective on mitochondrial disease. Lancet Neurol.

[2] Testai FD, Gorelick PB (2010). Inherited metabolic disorders and stroke part 1: Fabry disease and mitochondrial myopathy, encephalopathy, lactic acidosis, and strokelike episodes. Arch Neurol.

[3] Schaefer AM, Walker M, Turnbull DM, Taylor RW (2013). Endocrine disorders in mitochondrial disease. Mol Cell Endocrinol.

[4] Kubota H, Tanabe Y, Takanashi J, Kohno Y (2005). Episodic hyponatremia in mitochondrial encephalomyopathy, lactic acidosis, and strokelike episodes (MELAS). J Child Neurol.

[5] Aurangzeb S, Vale T, Tofaris G, Poulton J, Turner MR (2014). Mitochondrial encephalomyopathy with lactic acidosis and stroke-like episodes (MELAS) in the older adult. Pract Neurol.

[6] Nesbitt V, Pitceathly RD, Turnbull DM (2013 Aug). The UK MRC Mitochondrial Disease Patient Cohort Study: clinical phenotypes associated with the m.3243A>G mutation--implications for diagnosis and management. J Neurol Neurosurg Psychiatry.

[7] (2020). MELAS Syndrome - NORD (National Organization for Rare Disorders). https://rarediseases.org/rare-diseases/melas-syndrome/.

[8] Chinnery PF, Elliott C, Green GR (200). The spectrum of hearing loss due to mitochondrial DNA defects. Brain.

[9] Malhotra K, Liebeskind DS (2016). Imaging of MELAS. Curr Pain Headache Rep.

[10] Goodfellow JA, Dani K, Stewart W (2012). Mitochondrial myopathy, encephalopathy, lactic acidosis and stroke-like episodes: an important cause of stroke in young people. Postgrad Med J.

[11] Pfeffer G, Majamaa K, Turnbull DM (2012). Treatment for mitochondrial disorders. Cochrane Database Syst Rev.

